# Supplementary material for: Hepatic arterial infusion is effective in patients with unresectable colorectal liver metastases refractory to standard systemic chemotherapy: A retrospective cohort study
Source: PLoS One. 2025 Nov 6;20(11):e0336230. doi: 10.1371/journal.pone.0336230 (PMC12591460; doi:10.1371/journal.pone.0336230)
Supplement: Attachment — Submitted filename: HAIC-FOLFOX-B_Response to Reviewers0731.docx [file pone.0336230.s003.docx]

July 31, 2025

Emily Chenette

Editor-in-Chief

*PLOS ONE*

Dear Editor-in-Chief

I, along with my coauthors, would like to resubmit the attached manuscript titled “Hepatic arterial infusion is effective in patients with unresectable colorectal liver metastases refractory to standard systemic chemotherapy: A retrospective cohort study” as an original article. The manuscript ID is PONE-D-25-22185.

We thank you and the reviewers for your thoughtful suggestions and insights, which have enriched the manuscript and produced a more balanced and better account of the research. We hope that the revised manuscript is now suitable for publication in your journal.

The manuscript has been carefully rechecked and appropriate changes have been made in accordance with the reviewers’ suggestions. The responses to their comments have been prepared and attached herewith. Changes in the manuscript are shown in marked-up copy of my manuscript shown in “Track Changes” option in Microsoft Word., and a clean copy of the manuscript has also been attached.

Thank you for your consideration. I look forward to hearing from you.

Sincerely,

Osamu Itano, M.D., Ph.D.

Department of Hepato-Biliary-Pancreatic and Gastrointestinal Surgery, International University of Health and Welfare School of Medicine, 852 Hatakeda, Narita, Chiba 286-8686, Japan;

Email: laplivertiger@gmail.com

Telephone: +81-476-35-5600

Fax: +81-476-35-5586.

**Our point-by-point responses to the reviewers’ comments** **and suggestions are listed below:**

***Comments to the Author***

**Reviewer #1:** Main issues：
1.Inadequate Patient Allocation Justification：No explanation for the non-randomized assignment to HAIC-FOLFOX-B (n=20) vs. HAIC-FOLFIRI-B (n=22). Clarify selection criteria (e.g., physician discretion, tumor characteristics) to address potential selection bias.

***Response:***

We thank you for the suggestion. Accordingly, we explained the reason in the revised manuscript.

Page 9 line 129-130

Regarding patient allocation, the physician in charge considered the previously administered regimen and decided the regimen to be selected.

***Comments to the Author***

2. Contradictory Efficacy Conclusions：Abstract claims HAIC-FOLFOX-B is effective (RR 25%), yet results show inferior median OS (12.9 months) vs. HAIC-FOLFIRI-B (17.4 months). Reconcile this discrepancy and clarify clinical significance given non-significant p-values (OS: p=0.116; PFS: p=0.053).
***Response:***

We appreciate and agree with you on this comment. Abstracts showed HAIC-FOLFOX-B/ FOLFIRI-B regimens might be effective. We modified it as below.

Page 4 lines 47-49

HAIC FOLFOX-B and FOLFIRI-B regimens might be effective therapeutic options in patients with unresectable colorectal liver metastases refractory to standard systemic chemotherapy.

***Comments to the Author***
3.Incomplete Safety Reporting：Table 3 shows significantly higher anorexia in HAIC-FOLFIRI-B (18.2% vs. 0%, p=0.045) and artery occlusion (13.6% vs. 0%), yet these are not discussed. Expand safety analysis to address toxicity trade-offs between regimens.
***Response:***

We added a text to the Discussion section to expand it.
Pages 22-23 lines 272-285

In patients who underwent several systemic chemotherapies, assessing adverse effects was important. Although there were almost no differences in the adverse events following HAIC-FOLFOX-B and HAIC-FOLFIRI-B in this study, the patients who underwent HAIC-FOLFIRI-B experienced anorexia and artery occlusion more frequently than did the patients receiving HAIC-FOLFOX-B. The patients who underwent systemic FOLFIRI experienced anorexia more frequently than did the patients administered systemic FOLFOX [26]. Hepatic arterial occlusion has been reported to be the most frequent cause of interruption of HAIC [27]. Hamada et al. reported that almost 30% of patients who underwent HAIC experienced artery occlusion. [27] Our study showed that 7.1% of the patients who underwent HAIC had artery occlusion. Although this result was favorable, irinotecan might be likely to cause inflammation in the artery. The patients in this study experienced more anemia grade 3/4 than did those in other studies. However, many patients underwent several systemic chemotherapies before our treatment in this study. The bone marrow function of such patients might be possibly weakened.

***Comments to the Author***

4.Unclear Definition of "Refractory" Status：Lack of standardized criteria for prior chemotherapy failure (e.g., RECIST progression, cycles completed). Specify how "refractoriness" was determined to ensure patient homogeneity.

***Response:***

Thank you for your suggestions. We added the explanation to the revised manuscript.

Page 8 lines 105-107

All of these patients were refractory to both oxaliplatin-based and irinotecan-based chemotherapies. Refractory patients were defined as those who could not receive either regimen again due to adverse effects or disease progression.

**Reviewer #2:** This manuscript addresses an important clinical question about treatment options for patients with unresectable colorectal liver metastases refractory to standard systemic chemotherapy. The authors present a retrospective cohort study comparing two hepatic arterial infusion (HAIC) regimens combined with systemic bevacizumab. The topic is clinically relevant and the data are potentially useful for clinicians managing a difficult-to-treat patient population.
While the study is interesting, some major and minor issues should be addressed to improve its clarity, rigor, and presentation.
***Comments to the Author***
1. The manuscript mentions that all patients had previously failed systemic oxaliplatin-based and irinotecan-based therapies, but Table 1 shows heterogeneity in prior treatments (ranging from 2 to 7 prior lines). Please clarify how “refractory” was defined.

***Response:***

We added the explanation to the revised manuscript.

Page 8 lines 105-107

All of these patients were refractory to both oxaliplatin-based and irinotecan-based chemotherapies. Refractory patients were defined as those who could not receive either regimen again due to adverse effects or disease progression.

***Comments to the Author***
2. The p-values reported for OS and PFS between the two HAIC regimens are >0.05, yet the discussion sometimes implies superiority of one regimen over another.
***Response:***

Thank you for your comment. We added an explanation to the revised text.

Page 24-25 lines 317-320

According to this study, we found that HAIC-FOLFOX-B was suitable for use in the patients aiming for conversion surgery due to higher response rates. However, there was no difference in the survival between the HAIC-FOLFOX-B and HAIC-FOLFIRI-B groups.

***Comments to the Author***

3. Some toxicities (e.g., 16.7% grade 3–4 anemia, hepatic artery occlusion) are not trivial. Please clarify.

***Response:***

We appreciate and agree with you about the comment. We added an explanation to the Discussion section.

Page 22-23 lines 278-285

Hepatic arterial occlusion has been reported to be the most frequent cause of interruption of HAIC. [27] Hamada et al. reported that almost 30% of patients who underwent HAIC experienced artery occlusion. [27] Our study showed that 7.1% of the patients underwent HAIC had artery occlusion. Altough this result was favorable, irinotecan might be likely to cause inflammation in the artery. The patients in this study experienced more anemia grade 3/4 than did those in other studies. However, many patients underwent several systemic chemotherapies before our treatment in this study. The bone marrow function of such patients might be possibly weakened.

***Comments to the Author***

4. Figures are not visible in the PDF.
***Response:***

We added the figures again.

***Comments to the Author***

5. Table 4 is extensive but difficult to follow.
***Response:***

We omitted some of Table's lines.

| Regimen | | Author | Response rate (%) | | Median OS  since treatment  (months) | | |
| --- | --- | --- | --- | --- | --- | --- | --- |
| HAIC | Systemic chemotherapy |  |  |  |  |  |  |
| Pirarubicin | 5FU and LV | Fillik et al. [31] | 34.4 | | 20 | | |
| Cisplatin | 5FU | Macini et al. [32] | 52 | | 18 | | |
| Oxaliplatin | 5FU and LV | Ducreux et al. [33] | 64 | | 27 | | |
| 5FU and LV | 5FU and LV | Fiorentini et al. [34] | 47.5 | | 20 | | |
| 5FU and LV | UFT and LV | Tsutsumi et al. [35] | 87.5 | | 22 | | |
| Irinotecan, 5FU, and LV | UFT, and LV | Idelevich et al. [36] | 65 | | 36 | | |
| 5FU | Irinotecan and oxaliplatin | Kemeny et al. [37] | 100 (first line)  85 (prior chemotherapy) | | 51 (first line)  35 (prior chemotherapy) | | |
| 5FU | Irinotecan/oxaliplatin, 5FU, and Bev | D`Angelica et al. [38] | 76 | | 38 | | |
| Irinotecan, oxaliplatin, and 5FU | Cetuximab | Lévi et al. [39] | 40.6 | | 25.5 | | |
| Oxaliplatin | 5FU and LV,  or 5FU and Bev, or 5FU and anti EGFR therapy | Lim et al. [40] | 21.3 | | 13.5 (first and second line)  8.3 (third and fourth line) | |  |
| 5FU | Irinotecan/oxaliplatin and Bev or 5FU/LV/irinotecan and Bev | Pak et al. [41] | 73 | 38 | |  |  |
| 5FU, irinotecan/oxaliplatin | LV and Bev | Ishii et al. (current study) | 25/4.5 (third or more) | 12.9/17.4 (third or more) | |  |  |

***Comments to the Author***

6. The abstract does not specify that the study was retrospective or single-center.

***Response:***

We added a sentence to the abstract to specify the study design.

Page 3 lines 31-32

This study was a retrospective analysis conducted at a single center.
